# Public Trust in Health Information Sharing: Implications for Biobanking and Electronic Health Record Systems

**DOI:** 10.3390/jpm5010003

**Published:** 2015-02-03

**Authors:** Jodyn Platt, Sharon Kardia

**Affiliations:** 1 Department of Health Management and Policy, University of Michigan School of Public Health, 1415 Washington Heights, Ann Arbor, MI 48109, USA; 2 Department of Learning Health Sciences, University of Michigan Medical School, 1111 E. Catherine Street, Ann Arbor, MI 48109, USA; 3 Department of Epidemiology, University of Michigan School of Public Health,1415 Washington Heights, Ann Arbor, MI 48109, USA; E-Mail: skardia@umich.edu

**Keywords:** trust, biobanks, health systems

## Abstract

Biobanks are made all the more valuable when the biological samples they hold can be linked to health information collected in research, electronic health records, or public health practice. Public trust in such systems that share health information for research and health care practice is understudied. Our research examines characteristics of the general public that predict trust in a health system that includes researchers, health care providers, insurance companies and public health departments. We created a 119-item survey of predictors and attributes of system trust and fielded it using Amazon’s MTurk system (*n* = 447). We found that seeing one’s primary care provider, having a favorable view of data sharing and believing that data sharing will improve the quality of health care, as well as psychosocial factors (altruism and generalized trust) were positively and significantly associated with system trust. As expected, privacy concern, but counterintuitively, knowledge about health information sharing were negatively associated with system trust. We conclude that, in order to assure the public’s trust, policy makers charged with setting best practices for governance of biobanks and access to electronic health records should leverage critical access points to engage a diverse public in joint decision making.

## 1. Introduction

Health systems across the U.S. are investing in both electronic health record systems and institutional biobanks to expand research and accelerate improvements in personalized health care. The coupling of institutional biobanks with electronic health record systems make each resource more valuable since stored biological samples can be studied along with health information collected in health records or during the course of research. Although health data has been shared between diverse organizations (public health, research, healthcare, and insurance agencies) for decades, the quantity, quality, and rate of sharing has been propelled to new levels through advances in fields such as genomics. For example, the National Institutes of Health-sponsored Electronic Medical Records and Genomics (eMERGE) Network is increasing the integration of genomic data into medical records to accelerate the translation pipeline. The potential cost-efficiency of linking electronic health record (EHR) systems with biobanks is leading to the replication and expansion of networks able to share health information among a wider range of users and for a larger proportion of the public [1,2,3,4].

The idea that data and samples are collected for unknown future research projects strains current informed consent and data sharing models, which apply to discrete uses and emphasize the articulation of risks and benefits of specific research projects. In short, consent and data sharing operate on a one-form one-study model, while biobanking seeks to obtain consent and permission for data sharing for future research and/or for multiple projects. With a greater proportion of the population being represented in biobanks or in electronic health record systems, the risk to personal autonomy and to privacy necessarily increases in scale and scope. The policy and practice changes that biobanks and large health information systems demand transform systems of accountability and oversight as well as the terms and conditions for public trust, which is broadly recognized as critical to the ethical efficiency and practicability of highly networked systems [5].

However, shifting data acquisition from a cohort-driven approach to one that leverages the convenience and cost-efficiency of electronic health record data is not without its ethical, legal, and societal challenges, which are reflected in debates about electronic security, privacy, anonymity and identifiability, and benefit sharing [6,7,8,9,10,11,12]. Informed consent in particular struggles to meet the demands of increased data sharing and indefinite research use. Implementing “dynamic” [13,14] and “durable” [1] consent policies will require changes in how and when patients learn about research participation and their rights and responsibilities therein. To attain durable consent that meets the ethical demands of informed consent—autonomy, beneficence, and justice—mechanisms for establishing “access points” [15] are critical for gaining and maintaining an active relationship between patient, provider, and researcher. An access point is a direct, interpersonal interaction in which one of the individuals represents a complex system or institution and can thus serve to build and maintain trust. Access points mitigate the uncertainty of being involved in large, abstract systems through the direct and human interaction that forms the basis of a trusting relationship [15]. If an individual has the opportunity to interact with another who can represent organizational and institutional interests, the basis for trust (or mistrust) becomes tangible. Complex initiatives such as eMERGE and others that link electronic health record systems to biobanks used for public health and scientific research can generate public skepticism and doubt in both enterprises [15,16] thus driving an increased need for trust.

Trust relationships in health information systems may operate one-on-one, locally to a specific organization, or across institutional boundaries. The trustor to trustee relationship may involve any combination of individuals, organizations, institutions, or systems. Examples include the doctor-patient relationship, the relationship between consumers and an organization such as Kaiser Permanente or a local research university, and the relationships defined by agreements between health care providers and public health departments to report certain types of data and information. In advancing a vision for health information infrastructure in the United States, the Institute of Medicine describes the importance of a strong “fabric of trust” between stakeholders [5], including the general public, to assure the good intentions of data sharing for individuals, organizations, and institutions in providing health care services and conducting research. Most studies of trust in the health care context have evaluated the doctor-patient relationship. Relatively few have examined public perspectives of the system as a whole as the boundaries between research and practice become more fluid.

Empirical studies generally define trust as a cognitive expectation or willingness to impart authority and accept vulnerability to another in the fulfillment of a given set of tasks. A number of factors can influence the capacity and inclination to trust including the trustor’s past experience or willingness to trust on the one hand, and the trustee’s competency, reliability, reputation, honesty, or interestedness in the trusted relationship on the other. The trustor or trustee can be an individual, organization, or institution; the consequence of a trustee betraying confidence can mean revealing information to the wrong person, financial waste, or endangering lives [11]. Mediators and moderators of trust include characteristics of the relationship between trustor, trustee, and the context influencing the expectations and willingness to accept vulnerability [17,18,19,20,21,22,23]. In the present analysis, we focus on characteristics of the trustor that would influence their trust in an expanding health information system, which would underlie advances in personalized medicine.

### 1.1. Conceptual Model

**Figure 1:**
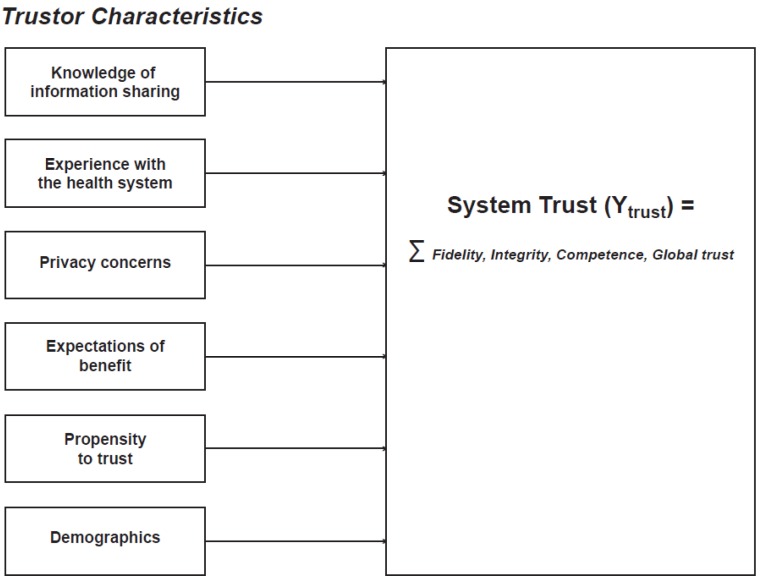
Conceptual Model.

To guide our investigation of the factors underlying the public’s trust in health information systems we created a conceptual model (Figure 1) representing six arenas anticipated to influence System Trust. Briefly, this model extends current research (reviewed below) on trust in the health system by measuring trust at the individual and system levels, examining four key dimensions of trust: Fidelity, competency, integrity, and global trust. It evaluates the relationship between trust in the health system and: (1) knowledge of health information sharing; (2) experience with the health system; (3) attitudes and beliefs about privacy; (4) expectations of benefit; (5) psychosocial factors; and (6) demographic characteristics.

### 1.2. System Trust and Its Dimensions

Surveys of trust in the health system encompass several dimensions most frequently including: Communication, honesty, confidence, competence, fidelity, system trust, confidentiality and fairness [24]. Hall *et al.* developed the Wake Forest Scale that has been applied to a number of relevant dimensions of the health system at large including trust in physicians [18,25], the medical profession [26], and insurance companies [27]. Other scales of trust in the health setting, organizations, and technology use dimensions that are consistent with the Wake Forest Scales (See e.g., LaVeist *et al*. on race, trust, and health [28]; McKnight, Choudhury, and Kacmar on technology [29]; and Siegrist on GMOs [30]). Our study focuses on fidelity, integrity, competency, and global trust dimensions as critical aspects of trust in information sharing necessary to implementing and scaling personalized medicine to large populations. Specifically, fidelity captures attitudes about the benevolence of the health system, *i.e.*, the ability of the system to prioritize the needs and interests of the public [31]. Integrity, defined as honesty, captures confidence in upholding the principles of non-deception. Competency refers to the ability and expertise to minimize errors and achieve goals. Global trust is an integrative concept that captures an individual’s general perception of trustworthiness. It is meant to capture aspects of trust for which a rational basis is not necessary [18].

### 1.3. Knowledge of Health Information Sharing

Research on the public understanding of science has used qualitative and quantitative methods to evaluate the question of whether the lack of support for science is simply a question of a knowledge deficit. Evans and Durant showed that more knowledgeable individuals were more likely to support general science research, but were less likely to support controversial scientific endeavors such as human embryology [32], suggesting that science is not to be given a carte blanche in determining the limits of acceptable fields of investigation. Bruce Wynne’s study of Cumbrian sheep farmers strongly suggested that increased knowledge among the “lay” public does not necessarily translate into increased trust in the “expert” [33,34,35]. In expanding the networks for health information, public engagement is identified as the key mechanism for building trust and acceptance, often under the assumption that this interactional form can overcome “knowledge deficits”. In short, “It is assumed that more or better knowledge or improved communication will enhance receptivity to innovations” [36]. These studies exemplify research that shows that knowledge impacts support for science, sometimes positively and sometimes negatively. To this end, our study looks to see whether knowledge impacts trust in data sharing and if so, whether or not it increases support. We developed a set of fact-based questions to measure an individual’s knowledge about current, common policies and practices for data sharing among health care providers, insurers, researchers, and public health.

### 1.4. Experience with the Health System

Trust is likely to be influenced by the amount of direct experience an individual has with the trustee [37]. Prior experience with the actors in a complex system creates a type of awareness and understanding that helps make large and abstract systems accessible, reducing uncertainty and increasing trust [15,38]. Nonetheless, prior experience or familiarity with the object of trust only offers the possibility that an individual might come to trust without actually guaranteeing it [39]. Drawing on Luhmann’s theory that familiarity increases trust insofar as it reduces uncertainty [40], we assessed whether or not respondents had any contact with the health system either by seeing a primary health provider or having insurance.

### 1.5. Trustor Expectations

In the absence of direct knowledge about information use or experience with the health system, the public can still hold expectations for what the outcomes and benefits of the system will be. Various national and international reports [41,42,43], and direct-to-consumer (e.g., 23andMe, PatientsLikeMe) and private big data initiatives (e.g., Blue Health Intelligence) make the claim that expanding information infrastructure and making data sharing more efficient will improve the quality of health care and improve health. Understanding the public’s view of these goals and their general view of data sharing sheds light on the expectations they hold in entering into a relationship in which trust plays a central role.

### 1.6. Trustor Privacy Concerns

Technological advances have improved electronic data security immensely and privacy considerations have often been down-played since encryption, password protections, and firewalls reduce the risk of data resources being compromised [7]. Survey research evaluating the public’s concerns about privacy suggests that it is a high salience issue, but that it is unclear to what extent fears about discrimination or a violation of privacy precludes participation in biobanks, a comparable arena in which data is collected and stored for future research use [44,45]. There is some evidence that trust may increase if an individual is confident in the ability of a system to protect individual privacy [37].

### 1.7. Trustor Psychosocial Characteristics

Individual-level factors create world-views, embody social structures, and reflect the experience of everyday life. Based on these experiences, some individuals are more likely to exhibit trusting attitudes than others, regardless of the whether the context for trust is health-related or otherwise. Psychosocial factors such as self-esteem, optimism, self-efficacy, and a non-specific expectancy that people are reliable (*i.e*., general trust) are likely to reflect these generalized attitudes and beliefs [46]. In cases involving unfamiliar actors, psychosocial factors become a particularly relevant antecedent since the trustor has little on which to base his trust beyond these intrinsic characteristics [47].

## 2. Experimental Section

### 2.1. Questionnaire Development

We developed a 119-item survey to evaluate predictors of trust in the health system, broadly defined as a web of relationships among health care providers, departments of health, insurance systems, and researchers, System Trust. We focused on including the six trustor characteristics described above in the conceptual model (Figure 1) as well as additional questions about trust in specific institutions (health care providers, researchers, and public health), quality of experience, perceived control, and adequacy of policy oversight. Measures of the dependent variable—system trust—and the independent variables used in this paper were adapted from prior studies and contextualized for the health system [18,26,27,28]. Specifically, we used the California Health Foundation’s 2005 National Consumer Health Privacy survey [48] and methods used in risk analysis literature (see e.g., [49,50]) to develop measures of knowledge, experience, and expectation of benefit. Questions from the Medical Mistrust Index [51], and related studies of privacy of health information were adapted to assess privacy [48,52]. The Health Privacy Survey also informed questions about expectation of benefit and knowledge. Additional knowledge questions were developed by the research team based on its collective experience in conducting community conversations about biobanking in the state of Michigan [13,53,54]. Questions from the General Social Survey [55], the General Self-efficacy Scale [56] and the Rosenberg Self Esteem Scale [57] were used to evaluate psychosocial factors. The complete survey as it was administered is available online [58].

To estimate and control for potential bias in participant responses due to the type of scale, we measured beliefs about privacy, psychosocial factors, and System Trust using two scales. Half of the participants were asked questions on a four-point bi-polar Agree/Disagree scale (Strongly Agree, Somewhat Agree, Somewhat Disagree, and Strongly Disagree). The other half responded to these questions using a four-point uni-polar scale based on the prompt: “How true are the following statements.” The value labels that followed were: Not at all true, somewhat true, fairly true, and very true. While there were some significant mean differences in the responses depending upon which scale was utilized, there was no difference in any of the regression relationships with System Trust. Consequently, we added a regression parameter to adjust for type of scale in all models presented here to correct for this potential bias but did not need to add interaction terms with the trustor characteristics being evaluated. Uniformly, the four-point unipolar scale had slightly better statistical properties in terms of its centering in the four point scale, including less skewness and kurtosis than the bi-polar scale.

### 2.2. Sample

In September 2013, we conducted an online Qualtrix survey of the general public (*n* = 447) using Amazon’s Mechanical Turk (MTurk) system. MTurk is an online Internet crowdsourcing marketplace that is increasingly being used for survey research and is a good source for efficiently gathering high-quality data [59]. MTurk workers are demographically at least if not more representative of the U.S. population as traditional subject pools taken from college undergraduate and Internet samples in terms of gender, race, age and education [60]. As compared to typical Internet convenience samples, non-response error seems to be less of a concern in MTurk samples.

### 2.3. Statistical Analysis

Descriptive distributional statistics were estimated on all variables to identify outliers or other distributional characteristics that may influence regression modeling. For the main outcomes of System Trust as well as four trustor characteristics (privacy, self-esteem, altruism, and self-efficacy), we used Chronbach’s alpha and principal component analysis to identify the most parsimonious set of survey questions that explained the most multivariate variation in that dimension. After removal of variables, new Chronbach’s alpha and principal components were estimated to confirm the reliability of the group of variables. Table 1 shows the Chronbach’s alpha estimations for the four dimensions of trust and four trustor characteristics (privacy, self-esteem, altruism, and self-efficacy) with the original set of variables and with the more parsimonious set derived from the principal component analysis. Supplementary Table 1, Table 2, Table 3 and Table 4 show the principal components and Eigenvectors for the variables in each of the four trust dimensions (fidelity, integrity, competency, and global trust), as well as the results of the PCA after variable removal.

**Table 1:** Chronbach’s Alpha for Indices.

Trust Dimension	All Variables		Reduced Set of Variables	
	No. of Items	Chronbach’s α	No. of Items	Chronbach’s α
Fidelity	8	0.792	6	0.665
Competency	9	0.816	6	0.699
Integrity	5	0.818	4	0.753
Global trust	4	0.915	4	0.915
Privacy	6	0.8278	5	0.7658
Self-esteem	6	0.9009	4	0.8452
Altruism	4	0.6915	4 (no change)	0.6915 (no change)
Self-efficacy	6	0.8966	4	0.8233

**Table 2:** Descriptive statistics of survey participants (*N* = 447) and univariate regression relationship with system trust.

Demographic Factor	Sample (%)	US Population ^a^ (%)	β' (Univariate)
Sex			
Male	51.5%	49.0%	Ref
Female	48.5%	51.0%	−0.16
Age			
18–25	21.3%	20.0%	Ref
26–34	40.0%	20.0%	−0.28
35–54	27.9%	30.0%	−0.27
55–64	8.05%	10.0%	−0.20
65+	2.68%	10.0%	0.30
Race/ Ethnicity			
White Non-Hispanic	76.1%	63.2%	Ref
Black Non-Hispanic	7.16%	12.9%	0.37
Asian Non-Hispanic	8.05%	5.2%	−0.06
Hispanic	4.70%	17.0%	−0.12
Other	3.13%	27.2%	0.17
Education			
High School diploma or less	12.5%	43.2%	Ref
Some college or 2-year college	42.1%	28.6%	−0.48
4-year college	36.9%	18.4%	−0.16
Masters or above	8.50%	9.8%	−0.21
Home ownership			
Owns home	37.6%		Ref
Does not own home	62.4%		−0.28
Self-rated health			−0.29**
Excellent	17.6%		
Very good	40.7%		
Good	28.6%		
Fair	11.4%		
Poor	1.57%		

** *p* < 0.05; ^a^ Data from U.S. Census Bureau, 2014.

Indices were then calculated for System Trust and key trustor characteristics (e.g., privacy index, esteem index, *etc.*) as the sum of the participant’s responses to those survey questions divided by the number of questions.

Ordinary Least Squares (OLS) Regression analysis was used to estimate the linear relationship between overall trust in the health system and each survey question and indices, (separately) before estimating a multivariable model using stepwise selection methods (inclusion criteria (*p* < 0.05) and backward elimination using exclusion criteria (*p* ≥ 0.10). In all models we included an indicator variable to control for whether a respondent answered questions using the bipolar or unipolar scale.

## 3. Results

### 3.1. Demographics

The descriptive statistics for our sample are displayed in Table 2. The participants were 51% male and 89.3% were under the age of 55 years old. The majority were non-Hispanic white (76.1%) but all major racial/ethnic groups were represented in the sample. Approximately 45% of participants had 4-years of college or more. Most of the survey participants rented or lived with family rather than owning their own home (62.4% *vs.* 37.6%). In terms of self-rated health, 40.7% reported being in excellent or very good health and only 13.0% reported being in fair or poor health. For general comparison, we included the values for these demographic variables that were available from the US Census 2012. While we did not perform formal statistical tests of comparison, it is clear to see that the MTurk sample is younger, less diverse, and more educated than the US population.

Examining the relationship between System Trust and the demographic variables, we found that only self-reported health status was significantly and negatively associated with the System Trust index (*β*' = −0.29; *p* = 0.003) such that those who reported being in better health were likely to trust the health system more than those in poor or fair health. All other demographic variables—sex, age, race/ethnicity, education, and homeownership—were not significantly associated with trust in the health system when examined individually.

**Table 3:** Trustor characteristics and univariate regression relationship with System Trust.

**Table 3a. Knowledge Questions**	**Correct Response**	**Mean (% Answered Correctly)**	**β'**	**R^2^**
State and local health departments collect information from physicians and clinics to monitor the health of communities	True	91.5%		
Permission is NOT required for research using your health information if your identity (name, address) has been removed	True	76.3%		
Your health information may be used in multiple studies without your permission or knowledge	True	72.0%		
Institutions may charge money to researchers to access health information	True	70.2%		
You own your health information	False	65.5%		
Your physician determines all uses of information in your medical record	False	64.2%		
A person’s permission is required for all health research	False	63.1%		
Researchers always need to obtain permission from you to access your medical record	False	48.5%		
Health insurance companies are prohibited from using your health information to deny your coverage	True	35.3%		
All forms of discrimination based on genetic information are prohibited by law	False	30.4%		
*Average total score (out of 10)*		6.17	−0.30 ***	0.11
**Table 3b. Experience**	**Frequency**	**%**	**β'**	**R^2^**
**Experience with primary care physician**				0.060
Does not have a primary care physician	141	31.5	Ref	
Has primary care physician but has not seen at least once in the past year	52	11.6	0.18	
Has primary care physician and has seen at least once in past year	254	56.8	1.0***	
**Experience with insurance**				0.036
Does not have health insurance	151	33.8	Ref	
Has health insurance, and has not had a gap in coverage in the past year	260	58.2	0.78***	
Has health insurance, and has had a gap in coverage in the past year	35	7.83	0.23	
**Table 3c: Beliefs about privacy**		**Mean (Range:1–4)**	**β'**	**R^2^**
Keeping my electronic personal health information private is very important to me		3.14	−0.18	0.006
I worry that private information about my health could be used against me		2.28	−1.0 ***	0.208
There are some things I would not tell my healthcare providers because I can’t trust them with the information		2.43	−0.82 ***	0.160
Doctors could share embarrassing information about me with people who have no business knowing		2.09	−0.93 ***	0.186
I believe the privacy of my electronic personal health information is seriously threatened		2.44	−0.85 ***	0.157
Privacy Index		2.48	−1.5 ***	0.246
**Table 3d: Expectations of health information sharing**		**Mean (Range:1–4)**	**β'**	**R^2^**
Given what you know about information sharing among organizations in the health system, do you generally have a favorable or unfavorable opinion?		2.75	1.85 ***	0.614
What effect do you think that health information sharing is likely to have on the quality of health care that you receive?		2.82	1.33 ***	0.305
How likely do you think it is that health information sharing will improve the health of people living in the United States?		2.51	1.17 ***	0.301
**Table 3e: Psychosocial factors**		**Mean (Range:1–4)**	**β'**	**R^2^**
**Self-esteem **				
I take a positive attitude toward myself		3.13	0.44 ***	0.036
I wish I could have more respect for myself		3.00	0.24 **	0.014
I feel that I have a number of good qualities		3.39	0.38 ***	0.019
All in all, I am inclined to feel that I am a failure		3.54	0.25 **	0.009
*Self-esteem index*		3.26	0.48 ***	0.027
**Altruism**				
I always find ways to help others less fortunate than me		2.75	0.56 ***	0.052
The dignity and well-being of all should be the most important concern in any society		3.14	0.40 ***	0.024
One of the problems of today’s society is that people are often not kind enough to others		3.07	−0.05	0.0009
All people who are unable to provide for their own needs should be helped by others		2.85	0.32 **	0.019
*Altruism Index*		2.95	0.56 ***	0.029
**Self-efficacy **				
I can manage to solve difficult problems if I try hard enough		3.23	0.56 ***	0.036
If someone opposes me, I can find the means and ways to get what I want		2.60	0.20	0.006
I am confident that I could deal efficiently with unexpected events		3.10	0.62 ***	0.051
I can solve most problems if I invest the necessary effort		3.27	0.48 ***	0.027
*Self-efficacy Index*		3.05	0.71 ***	0.041
**Optimism**				
I think the quality of life for the average person is getting worse, not better		2.72	0.58 ***	0.082
**Generalized trust**				
Generally speaking, most people can be trusted		2.45	0.90***	0.143

*** *p* < 0.001; ** *p* < 0.05; * *p* < 0.10.

**Table 4:** Multivariable stepwise regression analysis (Adjusted *R^2^* = 0.68).

Trustor Characteristics	β'	*p*-value
**Knowledge Questions**		
Average total score (out of 10).	−0.11	<0.001
**Experience**		
Has seen primary care physician and has seen at least once in past year.	0.34	0.002
**Beliefs about privacy**		
Privacy Index	−0.45	<0.001
**Expectations of health information sharing**		
Given what you know about information sharing among organizations in the health system, do you generally have a favorable or unfavorable opinion of it?	1.36	<0.001
What effect do you think that health information sharing is likely to have on the quality of health care that you receive?	0.22	0.007
**Psychosocial factors**		
**Altruism**		
I always find ways to help others less fortunate than me.	0.26	<0.001
**Generalized trust**		
Generally speaking, most people can be trusted.	0.22	<0.001

### 3.2. Knowledge of Health Information Sharing

We asked ten questions about common data sharing practices and policies to evaluate knowledge about health information sharing (See Table 3a). On average, individuals responded to 6 out of 10 correctly (CI: 6.00–6.37). Ranking from most correct to least correct, we found that most individuals (92%) responded correctly (“True”) to the statement “State and local health departments collect information from physicians and clinics to monitor the health of communities.” Most individuals responded incorrectly (*i.e*., only 30% answered correctly) to the question of whether all forms of discrimination based on genetic information are prohibited by law. We found that knowledge of information sharing was negatively associated with System Trust (*β*' = −0.30, *p* < 0.001) and explained 11% of the variation in System Trust.

### 3.3. Experience with the Health System

In our sample, 56.8% of survey participants reported having a primary care physician and having seen their primary care physician in the past year (See Table 3b). Nearly 60% had insurance without a gap in coverage over the past year. On average, having had experience with the health system by having and seeing a primary care physician was associated with a 1.0-point higher score on the trust index than not having a primary care physician at all. System trust among those that had a primary care doctor but had not visited in the past year was not significantly different from those who had no provider. Similarly, System Trust among those having insurance without a gap in coverage was significantly higher than the reference group (not having health insurance, *β*' = 0.78, *p* < 0.001). The difference in System Trust was not statistically significant between those who had insurance, but had had a gap in coverage and those who did not have insurance at all. Experience with a primary care provider and with having had health insurance variables explained 6% and 4% of the variation in trust in the health system respectively.

### 3.4. Privacy Concerns

Privacy concerns were negatively associated with trust in the health system (Table 3c). Of the five privacy questions included in the privacy index, being worried that health information could be used against a participant had the greatest effect size (*β*' = −1.00, *p* < 0.001). Using the privacy index as the predictor of trust, we found that a one-point increase in the privacy index was associated with a 1.5-point decrease in System Trust. We found that the privacy index explained more of the variation in System Trust (*R*^2^ = 0.246) than any of the individual questions.

### 3.5. Expectations of Benefit

Having a generally favorable opinion about information sharing in the health system, and believing that information sharing would improve the quality of health care and the health of people living in the U.S. were each positively associated with trust (*p* < 0.001) (Table 3d). In general, the mean of each of these variables was greater than 2.5 suggesting that most respondents hold a positive view of health information sharing and its’ potential to benefit health and health care by improving the health of the population in the U.S. or by improving quality of care. Having a favorable view of information sharing alone explained 61.4% of the variance in trust.

### 3.6. Psychosocial Factors

Psychosocial factors captured individual characteristics of self-esteem, altruism, self-efficacy, optimism, and generalized trust (Table 3e). Of the 14 psychosocial variables, all but two were significantly associated with trust: “One of the problems of today’s society is that people are often not kind enough to others,” a measure of altruism (*p* = 0.68), and, “If someone opposes me, I can find the means and ways to get what I want,” a measure of self-efficacy (*p* = 0.12). The self-esteem, altruism, and self-efficacy indices did no better at explaining the variation in System Trust than the individual variables. Optimism, captured by the response to the question, “I think the quality of life for the average person is getting worse, not better” explained about 8% of the variation in trust. Generalized trust explained about 14% of the variation in trust.

### 3.7. Multivariable Analysis

Demographic, knowledge, experience, privacy, expectation of benefit, psychosocial factors, and survey version variables were used in multivariable stepwise regression models to identify predictors of trust (see Table 4). Seven variables remained in the final model: Knowledge, having a primary care provider, the privacy index, 2 expectation of benefit variables, 2 psychosocial variables, and the variable indicating which version of the survey a participant was given. All were statistically significant (*p* < 0.01). Knowledge about data sharing practices, and having strong beliefs about the value of privacy were found to be negatively associated with trust. Having seen a primary care physician, having positive expectations for a benefit to health information sharing, and psychosocial factors (altruism, generalized trust) were found to have a positive association with trust. Having a generally favorable opinion about health information sharing had the greatest effect size (*β*' = 1.36, *p* < 0.001). The adjusted R^2^ for the multivariable model was 0.68, higher than the univariable models.

## 4. Conclusions

This study corroborates research that shows trust to be a multi-dimensional and complex interplay between the characteristics of the trustor, the trustee, and the context in which trust is negotiated. With its application in a world where electronic health records are replacing paper-based systems, and where these data systems are being built to maximize interoperability, the concomitant technical, ethical, and policy challenges have been discussed (See, e.g., [2,8,61]). Our study of trust sheds light on the factors that will be important in understanding the strength of the “trust fabric” of health information systems that increasingly integrate the power of biobanks with electronic health record systems. In examining characteristics of the trustor associated with trust in the health system, we found that knowledge, privacy, experience, expectations of benefit, and psychosocial factors are important in evaluating trust.

Knowledge and concerns about privacy were found to be the key factors in predicting lower levels of trust. With regard to knowledge, the results of this survey suggest that relying on the public to independently seek information or that simply informing the public of current practices may not automatically result in a more trusting environment; at present, to know information policy is not to love it, as is hypothesized frequently in the area of public support of basic science (*i.e.*, “to know science is to love it”) [34]. This finding provides an important caveat to community engagement research in the arena of biobanking and data sharing. Specifically, while engagement efforts have often revealed that a more informed public is more trusting and supportive population biobanking efforts [53,54,62,63], it is more likely the process of engagement that drives the support and not the top-down bestowing of information. Understanding the importance of process in informing the public reveals the need for and value of investing in community consultation approaches that seek engagement and education via partnership models or deliberative democracy methodologies [64,65,66].

Privacy has been a growing concern for Americans over the past two decades with major implications for system trust and stakeholders involved in linking electronic health records and biobanks [67,68]. Indeed, balancing privacy interests with public health and health care interests in sharing health information will be an ongoing issue in seeking public trust. Addressing privacy concerns is a task will fall to the stewards and brokers of health information who can provide key access points for individuals to understand how health information is used and negotiate the terms of such use. Representatives of the health information system might be physicians, who are already known to be trusted agents, or academic researchers, who are less known, but are likely to be trusted agents given the high profile of their universities in creating local identity and communities. Engaging these professional groups to evaluate to what extent they are willing and able to take on this added brokerage role will be important in developing trusted and trustworthy systems that bridge research and health care practice.

Factors associated with increased trust include having experience with a primary care provider, expectation of benefit and psychosocial factors. Not only having a primary care provider, but also visiting that provider on one or more occasions within the year, predicts trust in the health system. This suggests that inter-personal relationships can have a positive effect on trust building in complex systems. As the health system becomes increasingly interconnected in the electronic space, it will be critical to find mechanisms and spaces in which trust can be negotiated and built person-to-person as it is in the doctor-patient relationship.

Trust is further shaped by the quality, length, and nature of the relatedness of the trustor and trustee [23]. Notably, simply having a primary care provider, but not accessing that service, did not have any effect on system trust in our study. While a slow process, building trust at the provider level is likely to have positive spillover effects on other institutions in the health information system including research, public health, and insurance. Initiatives aimed at adding recruitment to “informed cohorts” [1] to patient-provider interactions are likely to have a positive impact on trust building and the feasibility of “durable consent.” At the same time, psychosocial variables indicating altruism and generalized trust remained statistically significant in the final multivariable model. These are opinions and beliefs that are likely important to understanding the trustor’s proclivity toward having a positive view of the health system, its institutions and organization, and its capacity to share health information in the best interests of the patient, the research participant, or the client. Many of these factors are intuitively understood in patient-provider relationships and in one-on-one discussions. Assuring access points in which dialogue about the relationship between biobanking and electronic health records is a two-way interaction, flexible to interpersonal dynamics, will further make consent a meaningful process.

One of the strongest predictors of system trust was having a positive view of data sharing. As a part of patient engagement or biobank recruitment, being able to demonstrate that health information sharing improves health care quality will be key in building public trust. Efforts to gauge overall opinion about health information sharing will shed critical insight into the state of trust in the health system. Stakeholder engagement, which has been a key component of biobanking in the past decade and has been integral to the more recent eMERGE Network, has informed technical design solutions and has been important to facilitating organizational change efforts [67]. These efforts should continue to identify the public’s expectations to understand how they might be met or betrayed in EHR and biobanking programs. Indeed, “durable consent,” will require trusting relationships [13,14,69] and implementation of policies and procedures that increase transparency, assure the protection of privacy, and demonstrate trustworthiness by stating how data sharing improves health and the quality of health care.

Future studies should examine system trust and its predictors using nationally representative samples. Our results thus far suggest that decision-makers in health information sharing need to explore mechanisms and policy options that effectively build or sustain trust as they develop partnerships to work across systems. Expanding networks of health information to include electronic health record systems and research biobanks promises to accelerate the slow road of translating research into personalized medical practice, but it remains to be seen what the explicit benefits are, to whom they accrue, and what and how the public sees these benefits distributed. Finding ways to build trust can help to harmonize systems of accountability and oversight more efficiently. This is a challenging task that may require substantial short-term investment with benefits that are hard to quantify. However, a policy of no change, which assumes trust and implicitly masks many of the current health information sharing practices, may be the most risky proposition for assuring the public’s trust in the complex arena of personalized medical care.

## Supplementary Files

Supplementary File 1
