# Supplementary material for: Public Trust in Health Information Sharing: Implications for Biobanking and Electronic Health Record Systems
Source: J Pers Med. 2015 Feb 3;5(1):3–21. doi: 10.3390/jpm5010003 (PMC4384055; doi:10.3390/jpm5010003)
Supplement: Supplementary File 1 [file jpm-05-00003-s001.pdf]

Supplementary Materials

Prinicipal Component Analysis (PCA)

Supplementary Tables S1–4 show the Eigenvectors and principle components for the variables in each of the four trust dimensions (fidelity, integrity, competency, and global trust) used to measure system trust, as well as the results of the PCA after variable removal.

Table S1. Principle Component Analysis, Fidelity.

Fidelity					
All Variables			Reduced Set of Variables		
Component	Eigenvalue	Cumulative Proportion	Component	Eigenvalue	Cumulative Proportion
Comp1	3.411	0.426	Comp1	2.293	0.382
Comp2	1.152	0.571	Comp2	1.145	0.573
Comp3	0.872	0.680	Comp3	0.865	0.717
Comp4	0.710	0.768	Comp4	0.679	0.830
Comp5	0.601	0.843	Comp5	0.590	0.929
Comp6	0.462	0.901	Comp6	0.428	1.000
Comp7	0.433	0.955			
Comp8	0.359	1.000			

Fidelity: All variables								
Variable	Comp1	Comp2	Comp3	Comp4	Comp5	Comp6	Comp7	Comp8
~~trust_fid1~~	~~0.420~~	~~0.084~~	~~−0.073~~	~~0.197~~	~~−0.128~~	~~−0.856~~	~~0.049~~	~~−0.145~~
trust_fid2	0.386	0.025	−0.060	−0.603	−0.472	0.063	0.209	0.463
trust_fid3	0.370	−0.450	0.180	0.087	0.223	0.008	−0.656	0.375
trust_fid4	0.349	−0.487	0.138	0.185	0.307	0.151	0.686	−0.023
trust_fid5	0.332	0.343	−0.053	0.676	−0.374	0.388	−0.010	0.149
trust_fid6	0.175	0.505	0.794	−0.138	0.254	0.001	0.033	−0.007
~~trust_fid7~~	~~0.442~~	~~−0.066~~	~~−0.059~~	~~−0.256~~	~~−0.118~~	~~0.291~~	~~−0.228~~	~~−0.762~~
trust_fid8	0.282	0.419	−0.551	−0.123	0.632	0.080	0.002	0.143

Fidelity: Reduced set of variables						
Variable	Comp1	Comp2	Comp3	Comp4	Comp5	Comp6
trust_fid2	0.468	0.042	−0.085	0.627	−0.613	0.065
trust_fid3	0.471	−0.448	0.138	−0.044	0.188	−0.722
trust_fid4	0.454	−0.487	0.089	−0.124	0.247	0.687
trust_fid5	0.417	0.336	−0.097	−0.737	−0.400	−0.014
trust_fid6	0.239	0.516	0.771	0.132	0.251	0.041
trust_fid8	0.349	0.426	−0.602	0.172	0.552	−0.018

Table S2. Principle Component Analysis, Integrity.

Integrity					
All Variables			Reduced Set of Variables		
Component	Eigenvalue	Cumulative Proportion	Component	Eigenvalue	Cumulative Proportion
Comp1	2.964	0.593	Comp1	2.335	0.584
Comp2	0.728	0.738	Comp2	0.723	0.765
Comp3	0.499	0.838	Comp3	0.492	0.887
Comp4	0.455	0.929	Comp4	0.450	1.000

Table S2. *Cont.*

**Integrity**					
**All Variables**			**All Variables**		
**Component**	**Component**	**Component**	**Component**	**Component**	**Component**
Comp5	0.355	1.000			

**Integrity: All variables**					
**Variable**	**Comp1**	**Comp2**	**Comp3**	**Comp4**	**Comp5**
trust_int2	0.4688	−0.004	−0.563	0.5113	0.4492
~~trust_int3~~	~~0.4952~~	~~−0.0449~~	~~−0.1466~~	~~0.1271~~	~~−0.8456~~
trust_int4	0.373	0.8359	0.3884	−0.0014	0.1065
trust_int5	0.4242	−0.53	0.6828	0.1943	0.1874
trust_int6	0.4647	−0.1353	−0.2108	−0.8274	0.1915

**Integrity: Reduced set of variables**				
**Variable**	**Comp1**	**Comp2**	**Comp3**	**Comp4**
trust_int2	0.533	−0.012	−0.571	0.624
trust_int4	0.438	0.819	0.370	−0.020
trust_int5	0.488	−0.552	0.654	0.170
trust_int6	0.535	−0.155	−0.331	−0.762

Table S3. Principle Component Analysis, Competency.

**Competency**					
**All Variables**			**Reduced Set of Variables**		
**Component**	**Eigenvalue**	**Cumulative Proportion**	**Component**	**Eigenvalue**	**Cumulative Proportion**
Comp1	3.708	0.412	Comp1	2.439	0.407
Comp2	1.637	0.594	Comp2	1.100	0.590
Comp3	1.036	0.709	Comp3	0.927	0.744
Comp4	0.737	0.791	Comp4	0.615	0.847
Comp5	0.565	0.854	Comp5	0.528	0.935
Comp6	0.533	0.913	Comp6	0.392	1.000
Comp7	0.337	0.950			
Comp8	0.271	0.980			
Comp9	0.177	1.000			

**Competency: All variables**									
**Variable**	**Comp1**	**Comp2**	**Comp3**	**Comp4**	**Comp5**	**Comp6**	**Comp7**	**Comp8**	**Comp9**
~~trust_comp1~~	~~0.4036~~	~~0.0174~~	~~−0.2979~~	~~−0.2727~~	~~−0.2123~~	~~0.4502~~	~~0.0134~~	~~0.6515~~	~~−0.036~~
trust_comp2	0.3504	−0.3958	−0.0319	0.1796	−0.2751	−0.2754	0.7285	−0.0579	0.0403
trust_comp3	0.4227	−0.0189	−0.2062	−0.3316	−0.2026	0.2508	−0.182	−0.7285	0.0279
~~trust_comp4~~	~~0.3617~~	~~−0.3451~~	~~0.0913~~	~~0.0305~~	~~−0.1459~~	~~−0.5484~~	~~−0.6161~~	~~0.1872~~	~~0.0643~~
trust_comp5	0.2976	−0.3007	−0.2467	0.4632	0.6857	0.2518	−0.0778	−0.0475	−0.061
trust_comp6	0.3137	0.5334	0.005	0.2843	−0.0893	−0.1834	0.0026	−0.0286	−0.7027
~~trust_comp7~~	~~0.2926~~	~~0.5653~~	~~−0.0313~~	~~0.3012~~	~~0.0048~~	~~−0.0885~~	~~0.0215~~	~~0.0166~~	~~0.7033~~
trust_comp8	0.1862	−0.1078	0.8135	0.2345	−0.1672	0.4526	−0.0633	−0.0079	0.0052
trust_comp9	0.3124	0.1322	0.3683	−0.5843	0.5575	−0.2194	0.2142	0.0552	−0.009

**Competency: Reduced set of variables**						
**Variable**	**Comp1**	**Comp2**	**Comp3**	**Comp4**	**Comp5**	**Comp6**
trust_comp2	0.457	−0.447	0.101	0.001	−0.496	0.579
trust_comp3	0.509	0.000	−0.302	−0.195	−0.386	−0.680

Table S3. *Cont.*

Competency: Reduced set of variables						
**Variable**	**Comp1**	**Comp2**	**Comp3**	**Comp4**	**Comp5**	**Comp6**
trust_comp5	0.396	−0.542	−0.103	0.170	0.706	−0.108
trust_comp6	0.347	0.524	−0.374	0.644	0.032	0.223
trust_comp8	0.288	0.178	0.862	0.286	−0.006	−0.246
trust_comp9	0.415	0.447	0.072	−0.661	0.324	0.284

Table S4. Principle Component Analysis, Global Trust.

Global Trust					
**All Variables**			**Reduced Set of Variables (NO CHANGE)**		
**Component**	**Eigenvalue**	**Cumulative Proportion**	**Component**	**Eigenvalue**	**Cumulative Proportion**
Comp1	3.203	0.801	Comp1	3.203	0.801
Comp2	0.356	0.890	Comp2	0.356	0.890
Comp3	0.293	0.963	Comp3	0.293	0.963
Comp4	0.147	1.000	Comp4	0.147	1.000

Global trust: All variables				
**Variable**	**Comp1**	**Comp2**	**Comp3**	**Comp4**
trust_glob1	0.5134	−0.015	−0.5683	0.6428
trust_glob2	0.5194	−0.2018	−0.3676	−0.7446
trust_glob3	0.4786	0.8043	0.3479	−0.0559
trust_glob4	0.4875	−0.5588	0.6487	0.1712
trust_glob1	0.5134	−0.015	−0.5683	0.6428

Global trust: Reduced set of variables (NO CHANGE)				
**Variable**	**Comp1**	**Comp2**	**Comp3**	**Comp4**
trust_glob1	0.5134	−0.015	−0.5683	0.6428
trust_glob2	0.5194	−0.2018	−0.3676	−0.7446
trust_glob3	0.4786	0.8043	0.3479	−0.0559
trust_glob4	0.4875	−0.5588	0.6487	0.1712

© 2015 by the authors; licensee MDPI, Basel, Switzerland. This article is an open access article distributed under the terms and conditions of the Creative Commons Attribution license (http://creativecommons.org/licenses/by/4.0/).
